# Surgical Repair of Bulbar Urethral Strictures: Advantages of Ventral, Dorsal, and Lateral Approaches and When to Choose Them

**DOI:** 10.1155/2015/397936

**Published:** 2015-10-21

**Authors:** Krishnan Venkatesan, Stephen Blakely, Dmitriy Nikolavsky

**Affiliations:** ^1^Department of Urology, MedStar Washington Hospital Center, 110 Irving Street NW, Suite 3B-19, Washington, DC 20010, USA; ^2^Department of Urology, State University of New York Upstate Medical University, 750 East Adams Street, Syracuse, NY 13210, USA; ^3^Division of Urology, University of Colorado, 12605 E. 16th Avenue, Aurora, CO 80045, USA

## Abstract

*Objectives*. To review the available literature describing the three most common approaches for buccal mucosal graft (BMG) augmentation during reconstruction of bulbar urethral strictures. Due to its excellent histological properties, buccal mucosa graft is now routinely used in urethral reconstruction. The best approach for the placement of such a graft remains controversial. *Methods*. PubMed search was conducted for available English literature describing outcomes of bulbar urethroplasty augmentation techniques using dorsal, ventral, and lateral approaches. Prospective and retrospective studies as well as meta-analyses and latest systematic reviews were included. *Results*. Most of the studies reviewed are of retrospective nature and majority described dorsal or ventral approaches. Medium- and long-term outcomes of all three approaches were comparable ranging between 80 and 88%. *Conclusion*. Various techniques of BMG augmentation urethroplasty have been described for repairs of bulbar urethral strictures. In this review, we describe and compare the three most common “competing” approaches for bulbar urethroplasty with utilization of BMG.

## 1. Introduction

Buccal mucosa graft (BMG) is now routinely used in urethral reconstruction since its popularization by Burger et al. in 1992 in pediatric reconstruction [1] and subsequently by El-Kasaby et al. in 1993 for adult urethroplasty [2]. Its use in urethroplasty is arguably the gold standard for treatment of medium- and long-length strictures [3]. The first use of buccal mucosa in urethral reconstruction is attributed to Professor Sapezhko who by 1894 had performed 4 operations on humans [4, 5]. In 1941, Humby, a British surgeon, described using buccal mucosa in hypospadias repair [6]. The excellent histological properties of buccal mucosa were subsequently described by Duckett et al. [7]. In comparison to skin, buccal mucosa holds the distinct advantage of being hairless and accustomed to a moist environment. Moreover, it has a thicker epithelial layer, thinner lamina propria, and a greater density of capillaries with an abundance of Type IV collagen. All these qualities are thought to improve graft inosculation and survival after transplantation.

Various techniques of BMG augmentation urethroplasty have been described for repairs of bulbar urethral strictures. In this review, we describe and compare the three most common “competing” approaches for bulbar urethroplasty with utilization of BMG.

## 2. Technique

### 2.1. Indications

Before describing the approach to bulbar stricture in detail, it is important to reiterate the indications for the use of oral mucosa in urethral reconstruction. The authors follow a traditional algorithm, where bulbar strictures <2 cm in length can mostly be treated with excision and primary anastomosis, whereby strictures longer than 2 cm may require adjunct maneuvers and the use of graft tissue to augment the caliber of the urethra. These maneuvers may include augmented anastomotic urethroplasty typically used for strictures between 2 and 5 cm in length or, for longer strictures, “pure” urethral augmentation in order to establish a larger gauge urethra. The choice of where and how to augment the urethra is discussed here in further detail.

### 2.2. Dorsal Onlay

This technique was first described by Barbagli et al. in 1998 and involved circumferential bulbar urethral dissection, dorsal stricturotomy followed by augmentation of the stricturotomy by a penile skin graft (in the first 31 patients) or by BMG (in the last 6 patients) [8]. The key step of the procedure was “quilting” or spread-fixation of the graft on the tunica albuginea overlying the corpora cavernosa prior to suturing the edges of urethral mucosa to the edges of the graft. Even spread-fixation has a range of implementation techniques; some surgeons prefer a “traditional” manner of suturing through the graft to the underlying tunica albuginea, while others advocate for use of a biologic “glue.” The advantage of suture quilting the graft includes microfenestration of the graft resulting from the surgical needle, which may aid in allowing any trapped blood to escape, preventing hematoma under the graft, and increasing the likelihood of proper buccal mucosa engraftment. This maneuver is critical in fostering sufficient graft apposition to the well-vascularized tissue of the corpora cavernosa and minimizing the risks of graft contracture and pseudodiverticula formation.

One of the advantages of the dorsal approach is that it yields a relatively bloodless operation. This is because the bulbar urethra is eccentrically located in the corpus spongiosum, with only thin dorsal coverage by corpus spongiosum that requires incision. Another advantage of the dorsal approach is its versatility and applicability for strictures of any length and location. The dorsal stricturotomy in the bulbous urethra can be extended proximally towards the membranous urethra or distally into penile urethra if required by intraoperative findings without dramatically altering the plan for reconstruction. In the event that complete or near-complete obliteration is identified after committing to dorsal stricturotomy, several solutions are described. These include (a) excision of the obstructed segment and conversion to augmented anastomotic urethroplasty [9], (b) removal of ventral mucosal strip and addition of ventral BMG onlay [10], and (c) ventral stricturotomy and addition of elliptical ventral inlay [11].

One of the disadvantages of the original dorsal approach is the need to circumferentially mobilize the urethra. Kulkarni et al. addressed this with their modification where mobilization is undertaken unilaterally and carried just across the midline dorsally, preserving the lateral blood supply on the contralateral side [12].

There have been numerous studies examining the success of BMG bulbar urethroplasty over the last two decades, with a wide range of follow-up and varying definitions of success. The Société Internationale d'Urologie (SIU) with the International Consultation on Urological Disease (ICUD) published a systematic review of 66 studies, describing outcomes of a total of 934 patients after dorsal onlay urethroplasty with average follow-up of 42 months and mean success rates of 88.3% [13]. Soon after, Barbagli et al. published a long-term retrospective paper on the deterioration rate of augmentation urethroplasty [14]. In this study, only patients with follow-up of greater than 6 years were included, totaling 81 patients after dorsal onlay BMG urethroplasty. At a median follow-up of 111 months, the authors reported an 80.2% success rate, defined as requiring absolutely no further instrumentation including dilation. This compared to 81.5% and 83.3% for ventral and lateral onlay techniques, respectively, with similar lengths of follow-up. The overall conclusion drawn from these reviews is that no significant difference exists in recurrence rates between dorsal, ventral, and lateral approaches to bulbar urethroplasty [13, 14].

### 2.3. Ventral Onlay

The ventral “patch” onlay urethroplasty came to the forefront of urethral reconstruction in 1996 when, encouraged by the use of BMG in complex pediatric hypospadias repair, Morey and McAninch applied the graft to repair strictures of the bulbar urethra [15]. The authors describe direct saggittal ventral urethrotomy through the diseased bulbar urethra, followed by sewing of the graft to each edge of the native urethral mucosa. Subsequently, the corpus spongiosum is closed over the graft in a second layer and the bulbospongiosus muscle over this. While there is no separate tissue to which the graft can be “quilted,” the spongiosal closure typically incorporates a small “bite” of the graft, to increase proper apposition to the spongiosum that will provide its blood supply. The technique was introduced contemporarily with Barbagli's dorsal onlay technique, and the advantages and superiority of each have been the subject of intense debate ever since.

Proponents of the ventral onlay cite a straightforward approach, not requiring extensive circumferential mobilization and the technical demand of dorsal graft placement. This allows urologists who treat strictures only occasionally to still feel comfortable in performing urethroplasty for strictures that may not be amenable to excision and primary anastomosis. Moreover, the argument may be made that the thicker, ventrally placed corpus spongiosum provides a more robust vascular bed for buccal mucosa engraftment. Another anatomic consideration is specific location of the bulbar stricture. Patterson and Chapple, in a comparison of surgical techniques, note that, for very proximal bulbar strictures, ventral onlay poses a clear advantage in exposure and technique and is the appropriate choice [16]. Palminteri et al. also contend that ventral placement of BMG in bulbar urethroplasty has no significant impact on sexual quality of life and in fact improved most measures of sexual life, aside from postejaculatory dribbling [17]. An additional benefit is that the ventral approach is amenable to use in complex situations, including recurrent stricture [18], after radiation [19], and with adjunct maneuvers such as gracilis muscle flap coverage in particularly high risk, long segment strictures [20]. The ventral approach has also been used as a direct route to the dorsal aspect of the urethra, allowing preservation of bilateral vascular supports to the urethra [21].

Opponents of the ventral technique point to the need to make incision through the thicker ventral corpus spongiosum in order to reach the eccentrically located bulbar urethra, resulting in a bloodier operation. There is also a concern about increased risk of sacculation, diverticulum, or pouch formation, as well as more frequent irritative voiding symptoms and urine infection [22]. In their review of 11 series, Patterson and Chapple note several groups with higher incidence of sacculation or diverticulum formation with resultant worse postvoid dribbling in ventral onlays. They go on, however, to document that an equal number of series found no significant anatomic or clinical difference in these findings in comparing ventral or dorsal onlay [16]. What is ultimately evident is that, in experienced hands and with meticulous technique, these issues can be minimized; furthermore, the issue of sacculation seems dramatically higher in older series based on the use of skin, versus the more modern use of BMG [3].

This being said, there are certain disadvantages to the ventral approach. Several authors [23, 24] have noted finite incidence of urethrocutaneous fistulae after ventral stricture repair with BMG, which is essentially unheard of in the dorsal approach. Reiterating an advantage of the dorsal approach mentioned earlier here, the ventral approach is less versatile, as it does not lend itself to extension of the urethrotomy distally into the penis should intraoperative findings require it.

While the global definition of success varies, a common criterion in most if not all series is the patency rate. The International Consultation on Urological Disease (ICUD) reviewed techniques in management of anterior strictures and found the success rate of ventral onlay to range from 43 to 100%. The authors summarize these series, generating a total number of 563 patients treated at a mean follow-up of 34.4 months, yielding a mean success rate of 88.8%, comparable with dorsal onlay urethroplasty. A number of smaller series, including a recent prospective randomized study, have compared dorsal and ventral techniques and reached a similar conclusion to the ICUD group: that there is no significant difference in success rates based on graft placement [13, 25, 26].

### 2.4. Lateral Onlay

Lateral onlay BMG augmentation urethroplasty is described but not well established in the literature. It is utilized infrequently and this is reflected by its limited description in the literature. The procedure resembles the ventral onlay technique described above; however, the urethrotomy is made laterally after unilateral urethral mobilization. The graft is similarly sutured in place and the spongiosum is closed over the graft.

As described above, the various locations of the urethrotomy in substitution urethroplasty afford different benefits and can also result in varying consequences. The lateral urethrotomy was described by Barbagli et al. in 2005 [27]. This actually preceded the description of the modified dorsal onlay technique where dissection remains unilateral. In a similar vein to the one-sided dissection technique described by Kulkarni et al. [12], it was felt that eliminating circumferential dissection would help preserve the contralateral urethral blood supply. Furthermore, avoiding urethrotomy through the robust ventral spongiosum may decrease intraoperative blood loss.

While the advantages of one-sided dissection are shared with the modern dorsal onlay technique, several advantages are lost with a lateral onlay procedure. There is a stronger potential for sacculation and diverticulum formation. Additionally, the corpora cavernosa, which are used as a structured vascular bed in dorsal onlay urethroplasty, are not utilized in the same manner in the lateral technique. And while it may seem easier to carry lateral urethrotomy as compared to a dorsal urethrotomy proximally into the membranous urethra, there is no actual data to support the use of lateral onlay in this setting.

In both lateral and ventral onlay, the spongiosum is closed over the BMG. However, in the case of lateral closure, the spongiosum can be rotated dorsally to protect the suture line. Unfortunately, the lateral spongiosal tissue is not as thick and vascular and accordingly may serve as a lower-quality bed for buccal mucosa engraftment. Like ventral grafting, lateral onlay urethroplasty should not be utilized in repair of pendulous urethral strictures. Aside from the similar concerns for sacculation, there is also a conceptual concern for lateral curvature. This is not specifically documented in the literature, likely because it is a technique already not employed in this arena.

One study describes outcomes in 6 patients undergoing lateral onlay urethroplasty. The nonreintervention rate at a mean of 42 months was 83%. Keeping in mind the context of a small sample size and the retrospective nature of the analysis, the lateral technique was comparable to dorsal (85%) and ventral (83%) onlay techniques [27].

The lateral approach offers few advantages, and those too are largely outweighed by its own disadvantages and the advantages of the dorsal and ventral approaches. This technique should be used sparingly and reserved for special circumstances when intraoperative limitations compromise the ability to complete dorsal mobilization.

## 3. Complications

The complications of bulbar urethral augmentation relate ostensibly more to the surgery itself, rather than any specific technique, although, as discussed in each of the sections above, particular techniques may predispose patients to specific postoperative concerns. Complications can include wound and/or urine infection, urethrocutaneous fistula, perineal hematoma, blood loss requiring transfusion, or nerve injuries related to positioning. The overall incidence is low, and, in their series comparing these 3 approaches, Barbagli et al. noted no such complications amongst 50 patients [27].

## 4. Conclusion

Because the ventral, dorsal, or lateral placement of BMG is typically determined based on location and length of stricture and surgeon preference, comparative studies are limited. This review outlines the best available evidence supporting each technique. Aside from one randomized trial and one systematic review, the remainder of the studies referenced in this paper are retrospective reviews. While the best data suggest that patency outcomes are similar for each technique, appropriate patient selection is paramount to utilize the strengths of a given technique and avoid its shortcoming.
